# The Chromone Alkaloid, Rohitukine, Affords Anti-Cancer Activity via Modulating Apoptosis Pathways in A549 Cell Line and Yeast Mitogen Activated Protein Kinase (MAPK) Pathway

**DOI:** 10.1371/journal.pone.0137991

**Published:** 2015-09-25

**Authors:** Safia, Mohd Kamil, Pooja Jadiya, Saba Sheikh, Ejazul Haque, Aamir Nazir, Vijai Lakshmi, Snober S. Mir

**Affiliations:** 1 Department of Bioengineering & Biosciences, Integral University, Lucknow, Uttar Pradesh, India; 2 Laboratory of Functional Genomics and Molecular Toxicology, Division of Toxicology, CSIR-Central Drug Research Institute, Lucknow, Uttar Pradesh, India; 3 Department of Biochemistry, King George's Medical University, Lucknow, Uttar Pradesh, India; Indian Institute of Integrative Medicine, INDIA

## Abstract

The field of cancer research and treatment has made significant progress, yet we are far from having completely safe, efficient and specific therapies that target cancer cells and spare the healthy tissues. Natural compounds may reduce the problems related to cancer treatment. Currently, many plant products are being used to treat cancer. In this study, Rohitukine, a natural occurring chromone alkaloid extracted from *Dysoxylum binectariferum*, was investigated for cytotoxic properties against budding yeast as well as against lung cancer (A549) cells. We endeavored to specifically study Rohitukine in *S*. *cerevisiae* in the context of MAPK pathways as yeast probably represents the experimental model where the organization and regulation of MAPK pathways are best understood. MAPK are evolutionarily conserved protein kinases that transfer extracellular signals to the machinery controlling essential cellular processes like growth, migration, differentiation, cell division and apoptosis. We aimed at carrying out hypothesis driven studies towards targeting the important network of cellular communication, a critical process that gets awry in cancer. Employing mutant strains of genetic model system *Saccharomyces cerevisiae*. *S*. *cerevisiae* encodes five MAPKs involved in control of distinct cellular responses such as growth, differentiation, migration and apoptosis. Our study involves gene knockouts of *Slt2* and *Hog1* which are functional homologs of human ERK5 and mammalian p38 MAPK, respectively. We performed cytotoxicity assay to evaluate the effect of Rohitukine on cell viability and also determined the effects of drug on generation of reactive oxygen species, induction of apoptosis and expression of *Slt2* and *Hog1* gene at mRNA level in the presence of drug. The results of this study show a differential effect in the activity of drug between the WT, *Slt2* and *Hog1* gene deletion strain indicating involvement of MAPK pathway. Further, we investigated Rohitukine induced cytotoxic effects in lung cancer cells and stimulated the productions of ROS after exposure for 24 hrs. Results from western blotting suggest that Rohitukine triggered apoptosis in A549 cell line through upregulation of p53, caspase9 and down regulation of Bcl-2 protein. The scope of this study is to understand the mechanism of anticancer activity of Rohitukine to increase the repertoire of anticancer drugs, so that problem created by emergence of resistance towards standard anticancer compounds can be alleviated.

## Introduction

The ever evolving affliction of cancer is mounting its challenges on researchers and clinicians as the disease continues to impose immense amount of health burden on a devastating global scale. Significant understanding of its mechanistic cues has been achieved through research efforts that have now proven that this ailment finds a strong cause in altered communication between and within cells [1]. Thus far, effective non-surgical remedies against the disease include chemotherapy and radiation based treatment regimens. However, a number of potential anti-cancer therapies, based on molecules from natural origin, have exhibited promise in treating cancer while exerting minimal undesired effects (anemia, nausea and hair loss) and countering the challenge of drug resistance [2]. In addition to side effect and drug resistance, the cost of chemotherapy drug is also very high as compared to the natural compound from the medicinal plants.

Rohitukine (C16H19NO5; 5, 7-dihydroxy- 8-(3-hydroxy-1-methyl-4-piperidinyl)-2-methyl- 4H-chromen-4-one), isolated from *Amoora rohituka*, *Dysoxylum binectariferum* and *Schumanniophyton problematicum*, is known to possess anti-inflammatory, anti-implantation, anti-fertility, anti-proliferative and immunomodulatory properties [3]. However anticancer mechanism of action of Rohitukine is not known and as per our comprehension for the first time it has been evaluated in genetic model system of budding yeast as well as in lung cancer cells. Hundreds of yeast genes exhibit a link to human disease genes as nearly 30% of notorious genes involved in human diseases have yeast orthologs [4]. It is interesting to note that 47% of the yeast genes could be successfully humanized [5]. *S*. *cerevisiae* is also helping in revealing important aspects of many diseases such as neurofibromatosis type l, colon cancer [6].

We endeavored to specifically study Rohitukine in *S*. *cerevisiae* in the context of MAPK pathways as yeast represents the experimental model where the organization and regulation of MAPK pathways are best understood [7]. MAPK are evolutionarily conserved protein kinases that transfer extracellular signals to the machinery controlling essential cellular processes like growth, migration, differentiation, cell division and apoptosis. Therefore, mutation in any of the kinases of these pathways is directly linked to cancer [8]. It is, hence, prudent to focus further research efforts towards designing mechanism-based anti-cancer compounds that act on specific molecular targets linked with the etiology of the disease [9]. Hence kinase cascade presents novel opportunities for development of new cancer therapies designed to be less toxic than conventional chemotherapeutic drugs [10]. The studies were conducted employing genetic model system *Saccharomyces cerevisiae* as it has been usefully exploited for elucidating the anticancer therapy in association with exposure to 5-fluorouracil [11]. Yeast is also valued as a striking model for anticancer drug research [12] as it has proven helpful in uncovering the cellular targets of different drugs including precious anti-cancer drug KP1019 [13]. The budding yeast has five types of MAPK including: Fus3, Kss1, Smk1, Hog1 and Slt2. Slt2 is the MAPK of the cell wall integrity pathway and functional homolog of human ERK5 that are activated in response to growth factors and stress conditions [14]. Hog1 is functional homolog of mammalian p38 MAPK and is chiefly activated in response to osmotic stress [15].

The studies reported herein, make use of the genetic model system *S*. *cerevisiae* towards deciphering the effects of Rohitukine on all important process of cellular communication mediated by MAP kinase pathway, thereby affecting cellular survival and death via apoptosis. The study also investigates the effect of Rohitukine on apoptosis within human lung cancer cell line and explores the possible mechanisms involved via studies on important modulators of the process.

## Materials and Methods

### Extraction of Rohitukine

Rohitukine was isolated from stem of *Dysoxylum binectariferum* as described previously [16]. Briefly, air-dried stem bark of the plant was extracted with 95% ethanol and then concentrated by reduced pressure. It is further fractionated into four fractions (chloroform, soluble n-butanol, n-hexane and insoluble n-butanol fraction). From chloroform fraction, a known alkaloid rohitukine {5,7-dihydroxy-2-methyl-8- [4-(3-hydroxy-1-methyl)-piperidinyl]-4H-1-benzopyran-4-one)} was isolated by repeated column chromatography over silica gel and further purification by HPLCLC- 20AD using methanol solvent 55:45 v/v, flow rate 1.0 ml/min. The characterization of compound was performed using IR, NMR, mass, derivatization, and comparison with available literatures. The purity of rohitukine was 99.6% and yield was 1%.

### Yeast culture and maintenance

In present study, Wild Type strain BY4741 (MATa his3Δ1 leu2Δ0 met15Δ0 ura3Δ0) and knockout strain of *Slt2* and *Hog1* gene (gift from Dr. A. Chakrabarti and Dr. R. C. Meena from Defence Institute of Physiology and Allied Sciences, DRDO, India) were employed. The yeast cells were grown in YPD media (1% yeast extract, 2% bactopeptone, 2% glucose) as per the method described earlier [17].

### Determination of Minimum inhibitory concentration

Minimum inhibitory concentration (MIC) of drug was determined both spectrophotometrically (by measuring O.D. at 600 nm using multiwell microplate reader: Multi Skan, Thermo Scientific) and visually. Rohitukine was dissolved in Dimethyl sulfoxide. The MIC for Rohitukine was determined by plotting O.D. at 600 nm versus concentrations of drug (20μg/ml to 100μg/ml) [18]. The concentration at MIC of the drug was used in all experiments.

### Evaluation of growth inhibition by spotting assay

After the drug treatment growth inhibition of yeast cells was assessed by spotting assay. Cells were grown on standard yeast extract-peptone-dextrose (YPD) media. For Spotting assays, 5-fold serial dilutions in YPD media were prepared from exponentially growing culture of the different strains. 2μL of each dilution was then spotted onto YPD plate in absence and presence of drug [19].The growth differences were recorded following incubation of the plates for 24hrs at 30°C.

### Detection of reactive oxygen species (ROS) in budding yeast

The detection of reactive oxygen species was carried out by employing 2’ 7’ Dichlorofluoresceindiacetate (H2-DCF-DA; Cat. no.–D399; Invitrogen) staining as previously described with some modification [20]. Briefly, levels of ROS were measured after 24 hrs of drug treatment by adding 0.5μM of H2-DCF-DA to cells for 15 min in dark. Cells were washed thrice with 1X PBS. Fluorescence microscopy was performed using a Zeiss Axioplan-2 microscope using an excitation wavelength of 485 nm and an emission wavelength of 520 nm. ROS production was quantified using image J software (Image J, National Institutes of Health, and Bethesda, MD). A total of 50 cells from each group were quantified for fluorescence intensity and statistical significance was calculated with respect to untreated control group.

### Estimation of mitochondrial content employing MitoTracker Deep Red staining

To check the effect of drug on mitochondrial content, Mito Tracker Deep Red staining (Cat. no.-22426, Invitrogen) was done as described previously with some modifications [20]. Briefly, 100μl yeast cells were incubated with 100 nM Mito Tracker stain for 50 min at 30°C in dark followed by three times washing in 1X PBS. Imaging of cells was carried out using fluorescence microscope with an excitation wavelength of 637 nm and an emission wavelength of 660 nm. Fluorescence intensity of mitochondrial content was quantified using Image-J software.

### Assay for apoptotic cell death using Acridine Orange (AO) staining

Acridine orange staining was done to check the induction of early stage apoptosis. Acridine orange (Hi-media- 116) was dissolved in PBS (pH = 7). A 100μl volume of yeast cells was stained with 1μl of 2.5 mg/ml of AO to get the working concentration of 25μg/ml. Staining was carried out for 30 minutes in dark and the cells were washed with PBS [21]. Imaging of stained cells was carried out using fluorescence microscope with an excitation wavelength of 502 nm and an emission wavelength of 520 nm. Fluorescence intensity of stained cells was quantified by Image J software.

### Assay for studying DNA fragmentation

DNA fragmentation was determined by Nuc Blue Live Cell Stain (R37605 Life Technology Corporation) according to the manufacturer’s instructions. Imaging of stained cells was done by fluorescence microscope with an excitation wavelength of 352 nm and an emission wavelength of 460 nm and fluorescence intensity of stained cells was quantified by Image J software.

### Semi-Quantitative Reverse transcription PCR for the analysis of mRNA levels of *Slt2* and *Hog1* gene in the presence of Rohitukine

Total RNA was extracted and reverse transcribed using Revert Aid™ First Strand cDNA Synthesis Kit (Fermentas Life Sciences, cat- K1622). cDNA was amplified using specific primers listed in Table 1 and PCR products were separated on 1.5% agarose gel and visualized by ethidium bromide staining.

**Table 1:** Sequence of primers used.

Gene	Primer (5’-3’)	PCR Product Size (bp)	Annealing Temperature (°C)
ACT1	GCCATTTTGAGAATCGATTTG (F)	254	56
	TTAGAAACACTTGTGGTGAAC (R)		
SLT2	AGCAACAGCAGCCTTCAGA (F)	460	60
	GAACGCGAGGAAGTATCCAA (R)		
HOG1	ATTTGGGTTGGTTTGCTCAG (F)	254	54
	TTTCCAAGGGTCTTGTTTGC (R)		

### Protein-ligand interaction employing computational tools

The 3-dimensional (3D) structure of p38 and ERK5 used for docking study was retrieved from Protein data bank with PDB IDs: 1WFC and 4IC8 respectively. The structure of ligand (Rohitukine) (CID: 13422573) was accessed from ‘Pubchem compound’. Using the AutoDock tools Essential hydrogen atoms, Kollman united atom type charges, and solvation parameters were added. Affinity (grid) maps of 60×60×60 Å grid points and 0.375 Å spacing were generated using the Autogrid program aimed at targeting grid co-ordinates in proximity with the active site of targets. Accordingly, the values of x, y and z co-ordinates used for targeting the Hog1 and ERK5 active site were 18.783, 35.698, 30.394 for Hog1 and 15.928, -17.057, 1.101 for ERK5 respectively. Docking simulations were performed using the Lamarckian genetic algorithm (LGA) and the Solis & Wets local search method. Ten different runs were performed for each docking. The final figures were generated with the help of Discovery Studio Visualizer (Accelrys).

### Cell Culture

A549 cells (human lung cancer cell line) were obtained from National Centre for Cell Sciences (NCCS) Pune, India, and cultured in DMEM (Dulbecco’s Modified Eagle Media) F-12 (1:1) (HiMedia AL187A) supplemented with 10% fetal bovine serum, 0.2% sodium bicarbonate and 1% antibiotic and antimycotic solution. Cultures were maintained at 37°C and 5% CO2 and 95% humid atmosphere.

### MTT assay

MTT (HiMedia-TC191) assay is based on the reduction of MTT by mitochondrial dehydrogenase to a purple formazan product, gives an indication of mitochondrial integrity, which is interpreted as assessment of percent cell viability [22]. Briefly, cells were seeded in 96–well tissue culture plates (10^4^ cells/well) in complete DMEM F-12 medium, followed by incubation in 5% CO2, 95% atmosphere for 24hrs at 37°C. After 24hrs exposure of drug (10μM—60 μM), MTT (5 mg/ml of stock in PBS) was added (10 ml/ well in 100 ml of cell suspension), and plates were incubated for 4hrs. After incubation, the reaction mixture was carefully taken out and 200 μl of dimethyl sulfoxide (DMSO) was added to each well, the contents were mixed well by pipetting up and down several times. The plates were kept on rocker shaker for 10 min at room temperature and then read at 550 nm using multiwell microplate Reader (Multi Skan, Thermo Scientific). Untreated cells were run under identical conditions and served as basal control. Each experiment was repeated thrice and standard deviations were derived from three independent experiments.

### Determination of Reactive Oxygen Species (ROS) in lung cancer cells

ROS generation was estimated by using 2’, 7’-diclorodihydrofluorescein di-acetate (H2-DCF-DA; Cat. no.–D399; Invitrogen) as described previously [23]. Briefly, cells seeded in black 96-well plate at a density of 10^4^ cells/well were incubated with 1mM H2-DCF-DA; for 30 min at 37°C followed by incubation with different concentrations of drug for 24hrs. The measurement of ROS was carried out during the course of the treatment period at 485 nm excitation and 535 nm emission wavelengths. ROS generation was also confirmed by fluorescence micrograph of cellular ROS. Briefly, cells were plated in 48-well tissue culture plate and treated with 1mM H2-DCF-DA; for 30 min followed by incubation with different concentrations of Rohitukine for 24 hrs at 37°C. Fluorescence images were captured using a Zeiss Axioplan-2 microscope using FITC filter under 20X objective.

### Isolation of Total Cellular Protein from A549 cells

Rohitukine treated and untreated cells were pelleted, washed with cold PBS and lysed in RIPA lysis buffer containing 1 mM EDTA, 50 mM Tris, pH 7.4, 150 mM NaCl, 1% NP-40, 0.25% sodium deoxycholate, 0.1% SDS, 1 mM NaF, 1 mM Na_3_VO4, 1 mM PMSF and 1μg/mL leupeptin [24]. Cell lysate was gently vortexed for 30 sec after 1 h incubation in lysis buffer. Supernatant was collected by centrifugation at 14,000×g for 15 min and stored in aliquots at -20°C. Protein content was quantified using Bradford protein assay.

### Western Blot Analysis Detecting Apoptosis-related proteins

Cell lysates were denatured and twenty microgram of the protein was separated on 12% SDS–polyacrylamide gel electrophoresis. It was electro-transferred to PVDF membrane. The membranes were blocked at room temperature with 5% skimmed milk in Tris-buffered saline (TBS) with 0.05% Tween-20 (TBS-T) for 2hrs. After washing with TBS-T membranes were incubated with the primary antibodies against p53 (1:3000), caspase9 (1:3000), Bcl-2 (1:5000) and β-actin (1:4000) for overnight at 4°C. After washing, the membrane was incubated with HRP-conjugated secondary antibody (anti-rabbit or anti-mouse, 1:10000; Invitrogen, USA) at room temperature for 1h. Western blot bands were detected using chemiluminescent substrate (Millipore) using Chemidoc (GE). β-actin was used as internal control for equal loading and normalization of protein. Protein Ladder (3B BlackBio Biotech-3B75) (3.5–245kDa) was used to determine molecular weight of the protein bands. Densitometry of the bands obtained was done by NIH software Image J version 1.41 (USA).

### Statistical analysis

All results are presented as mean ± SEM Statistical significance between various groups was carried out employing Student’s t test by using Graph Pad prism 5 software. For *in vitro* study data were expressed as mean ±S.D. and statistical significance of the results were determined using one-way ANOVA by Tukey’s multiple comparison test.

## Results and Discussion

### ΔSlt2 and ΔHog1 strains are hypersensitive to Rohitukine treatment

**Fig 1:**
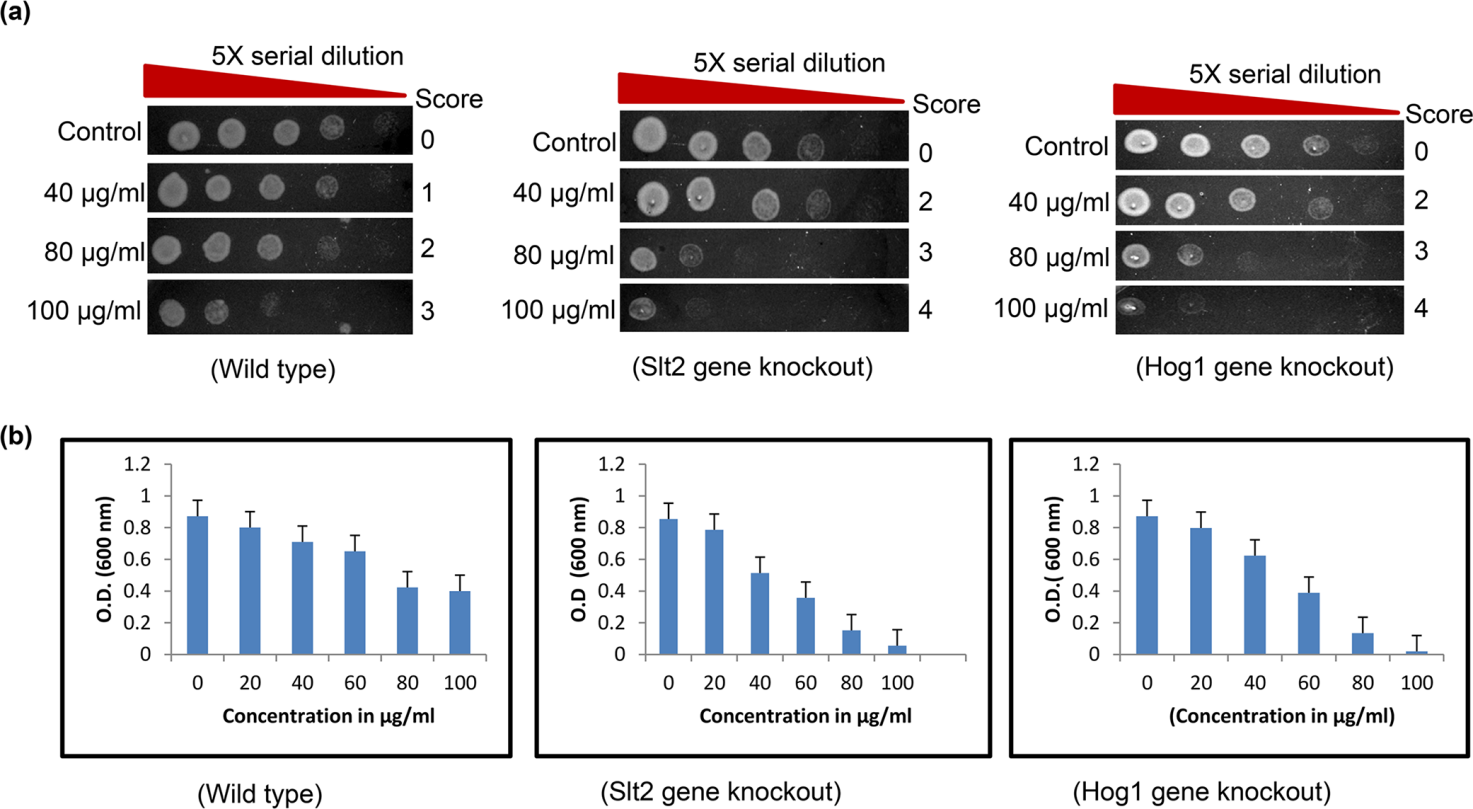
Hypersensitivity of ΔSlt2 and ΔHog1 strains to Rohitukine. (a) Yeast cells viability at different concentration of Rohitukine after 24 hrs of drug treatment, 5-fold serial dilutions from exponentially growing cultures of WT, ΔSlt2 and ΔHog1 strains were spotted onto YPD medium containing 40 μg/ml, 80 μg/ml and 100 μg/ml of drug. (b)The percentage of surviving cells relative to untreated controls.

In this study, we determined cytotoxicity of Rohitukine in budding yeast and also investigate whether MAP kinase pathways are involved in the Rohitukine induced cell death, so we determined the effect of Rohitukine on the cell viability of ΔSlt2 and ΔHog1 strains. Rohitukine shows cytotoxicity against all type of yeast strains. The MIC_50_ for Rohitukine was determined by plotting O.D. at 600 nm versus concentrations of drug. MIC_50_ value for WT was found to be 80μg/ml and for both gene knock-out strains was found to be 60μg/ml. All experiments were carried out at dose below the MIC_50_ value (40μg/ml). Fig 1 shows that Rohitukine exerts cytotoxic effects on yeast. Although the concentration of Rohitukine that kills approximately 50% of yeast cells is higher than the IC_50_ values reported for cancer cells in vitro [25] this result is not surprising given that yeast often display higher levels of resistance to antineoplastic agents [26] It is likely that the presence of yeast cell wall may be the obstruction of drug entry into cell and lots of export transporter that will interfere with the entry of drug inside the cell and yeast cells are also very effective at reducing intracellular concentration of toxic small molecules using a large number of transport proteins [27].

The results showed that gene knockout strains were more sensitive to the drug as compared to WT strain at same MIC (40μg/ml) as indicated by density of the spots in spotting assay for cell viability (Fig 1A) and by O.D at 600 nm (Fig 1B) Result of spotting assay confirmed that the yeast cells when treated with Rohitukine lost cell viability in dose dependent manner. In case of WT cells control spot was scored as 0, 40μg/ml of Rohitukine treated cells spot was scored as 1, cells were scored as 2 at 80μg/ml of drug and at 100μg/ml cells spot scored as 3 after 24 hrs of drug treatment. For both types of gene knockouts (Δslt2, Δhog1) strain control spot was scored as 0, 40μg/ml of drug treated cells scored as 2, 80μg/ml of drug treated cells scored as 3 and 100μg/ml of drug treated cells scored as 4 after 24 hrs of drug treatment. ΔSlt2 strain was hypersensitive to various genotoxic agents having different mode of action including methylmetanosulfonate, UV radiation and phleomycin [28]. Slt2 activation after induction of a single DSB (double-strand break) in the GAL1: HO strain, which has a specific effect on integrity of DNA, showing a genuine role for Slt2 in the response to genotoxic stress [29]. Consequently, these genes get activated in the presence of drug in WT cells so it could be possible that ΔSlt2 and ΔHog1 strains showed hypersensitivity to drug [30].

### Rohitukine triggers cell death by inducing oxidative stress and reducing mitochondrial content in ΔSlt2 and ΔHog1 strains

**Fig 2:**
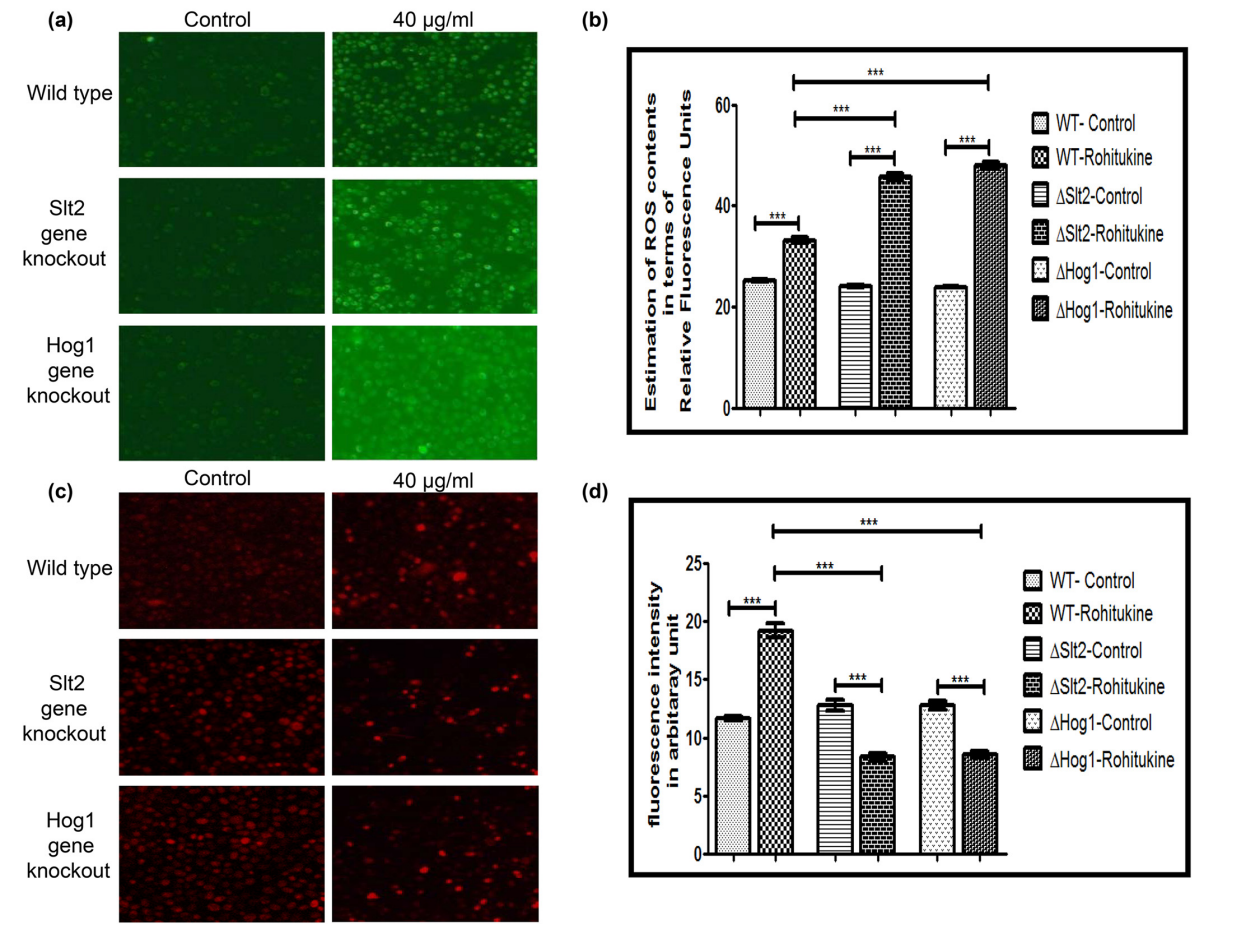
Rohitukine promoted ROS production and loss Mitochondrial content in gene knockout strains of yeast. (a) DCFDA staining (b) Graphical representation of Relative formation of reactive oxygen species (ROS) measured by H2DCFDA staining in WT and gene knockout strains of yeast as quantified using Image J software ***p < 0.001.(c) Mitotracker Deep Red staining (d) Graphical representation for fluorescence intensity of mitochondrial content of the budding yeast as quantified using Image J software ***p <0.001.

ROS production was measured to analyze the role of ROS in yeast cell death mediated by Rohitukine. We found that Rohitukine induced significant amount of ROS after 24 hrs of drug treatment in WT and in gene knockout strains (Fig 2A). Quantification of fluorescence intensity of H2-DCF-DA staining(Fig 2B) also showed that Rohitukine treated yeast cells produced comparatively increased levels of ROS as compared to untreated cells, increase being 1.3 (P<0.001), 2.0 (P<0.001) and 1.7 (P<0.001) fold for WT, ΔSlt2 and ΔHog1 strains respectively after Rohitukine treatment as compared to untreated control cells. However ΔSlt2 and ΔHog1 strains produced more ROS as compared to WT cells after treatment and increase being 1.4 (P<0.001) and 1.2 (P<0.001) fold for ΔSlt2 and ΔHog1 strains respectively as compared to WT after drug treatment, which further reinforces the hypersensitivity of both mutant strains to drug. This discrepancy may be because of absence of MAPK which is known to be activated by oxidative stress. Additionally MAPK deficient yeast cells accumulate ROS to a higher extent than WT cells during stationary phase [31]. MAP kinase pathways are influenced not only by receptor ligand interactions, but also by different stressors like oxidative stress induced potential activation of MAPK pathways. Generally, increased ROS production in the cells causes activation of MAPKs but the mechanisms by which ROS can activate these kinases are unclear [32]. These mutants may be impaired in autophagy pathway which is required to prevent excessive ROS accumulation. Inability to increase the expression of respiratory chain components and ROS scavengers likely leads to the accumulation of ROS in autophagy-defective cells. *S*. *cerevisiae* Hog1 MAPK is activated in response to high osmolarity and is required for cell survival under these conditions [33]. In response to several stresses, Hog1p becomes phosphorylated and translocates to the nucleus. Hog1 null mutants were found to be hypersensitive to those stress conditions, which lead to Hog1p activation, in particular to extracellular oxidizing agents [34].

We also checked whether Rohitukine exposure affects mitochondrial content. We observed that mitochondrial content decreased to a greater extent in ΔSlt2 and ΔHog1 strain after 24 hrs of Rohitukine treatment but WT cells showed increase in mitochondria content after drug treatment (Fig 2C). Quantification of fluorescence intensity of mitochondrial content (Fig 2D) showed that Rohitukine treated WT cells showing a 1.6 (P<0.001) fold increase whereas Rohitukine treated ΔSlt2 and ΔHog1 strains exhibiting 1.2 (P<0.001) and 2.0 (P<0.001) fold reduction respectively as compared to their untreated control. However, ΔSlt2 showed 2.3 (P<0.001) and ΔHog1 strain exhibited 2.2 (P<0.001) fold reduction as compared to WT in presence of drug.

The mitochondria also play a very important role in regulation of many mechanisms controlling cell survival and death [35]. Changes in mitochondria are related to aging, decreased synthesis of mitochondrial proteins and reduced activity of oxidative enzymes cause decrease in mitochondrial ATP synthesis [36]. MAP kinase pathways are involved in intrinsic apoptosis in the presence of isoorientin in human hepatoblastoma cancer cells [37]. Flavopiridol (Rohitukine derivative) causes cell death by decrease in mitochondrial membrane potential in human leukemia cells [38]. Flavopiridol also causes STI571 (Bcr/Abl kinase inhibitor) induced apoptosis and damage of mitochondria and apoptosis in BCR-ABL-positive human leukemia cells [39]. Consequently MAPK mutants show the increased production of ROS which maybe the likely cause leading to mitochondria dysfunction.

### Rohitukine causes induction of early stage apoptosis and DNA damage in yeast

**Fig 3:**
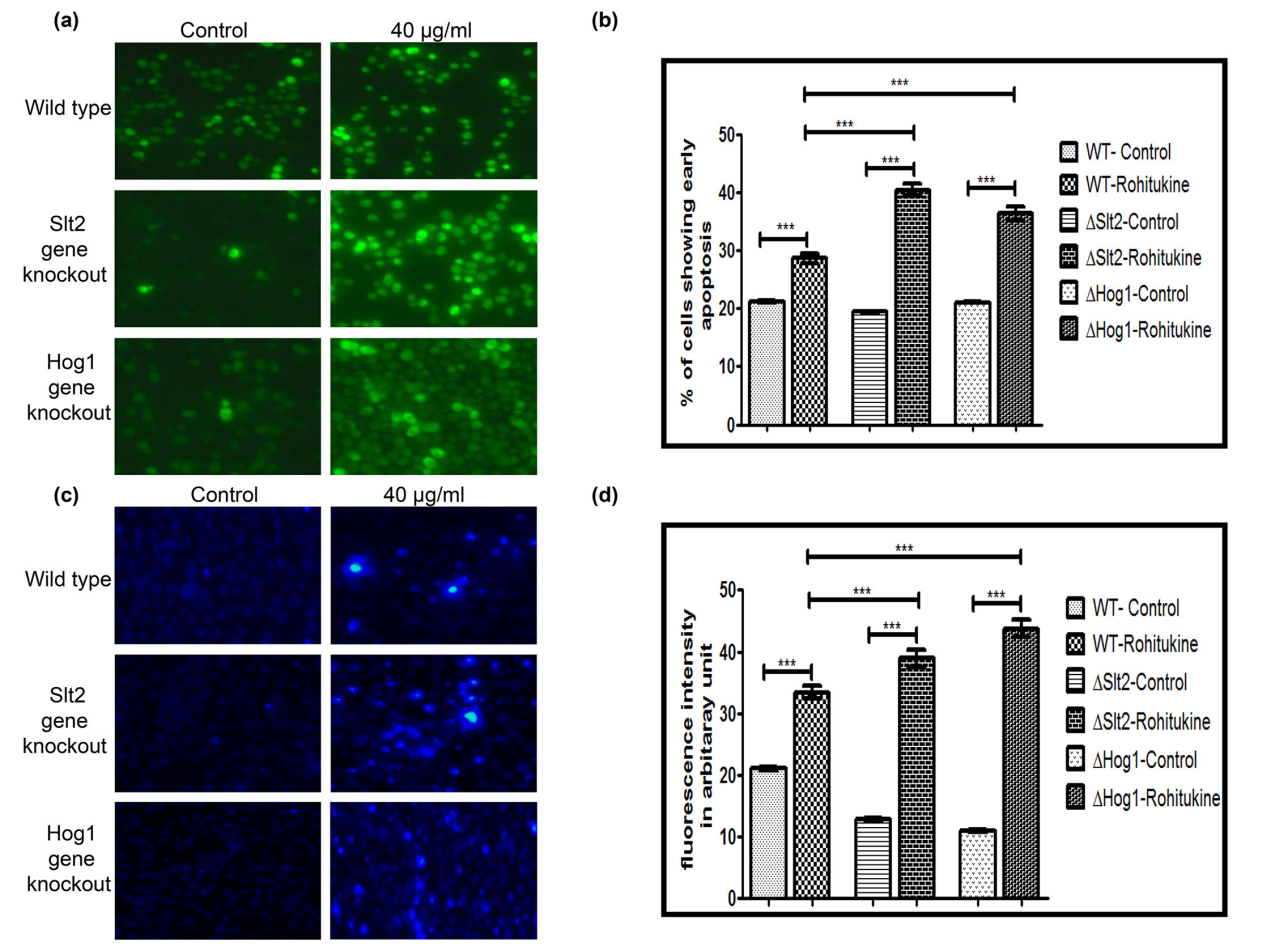
Rohitukine causes DNA damage and induction of apoptosis. (a) A.O. staining (b) Graphical representation for fluorescence intensity of apoptotic death of the Yeast cells as quantified using Image J software ***p < 0.001 (c) DNA damage revealed by Nuc Blue Live Cell Stain (d) Graphical representation for fluorescence intensity of nucleic acid of the yeast cells as quantified using Image J software ***p < 0.001.

Data of A.O staining showed that Rohitukine causes induction of apoptosis in gene knockout strains as compared to the WT strain after 24 hrs of drug treatment (Fig 3A). Quantification of fluorescence intensity of A.O staining (Fig 3B) showed that WT, ΔSlt2 and ΔHog1 stains of yeast exhibiting a 1.3 (p<0.001), 2.0 (p<0.001) and 1.7 (p<0.001) fold increase respectively after drug treatment as compared to their untreated control. However, ΔSlt2 strain showed 1.7 (p<0.001) and ΔHog1 strain exhibited 1.2 (p<0.001) fold increase as compared to the WT strain when treated with Rohitukine.

We also observed DNA fragmentation after 24 hrs of drug treatment at MIC by NucBlue Live Cell Stain for DNA. Result of DNA staining (Fig 3C) clearly showed DNA fragmentation in WT as well as in both types of gene knockout strains after drug treatment indicating an apoptotic phenotype. We quantified images for fluorescence intensity of DNA staining (Fig 3D). There was 1.5 (p<0.001), 3.0 (p<0.001) and 3.9 (p<0.001) fold increase in Rohitukine treated WT, ΔSlt2 and ΔHog1 strain respectively as compared to untreated control. However, ΔSlt2 showed 1.1 (p<0.001) and ΔHog1 strain exhibited 1.3 (p<0.001) fold increase as compared to the Rohitukine treated WT cells.

DNA fragmentation, a hallmark of apoptosis [40] was observed in all types of yeast strains after drug treatment as seen by DAPI staining. Valproic acid induces apoptosis by ROS generation and DNA fragmentation independent of Yca1p at concentrations that mildly affect the proliferation of yeast [41].

### Rohitukine interaction affects expression of *Slt2* and *Hog1* gene in wild type strain

**Fig 4:**
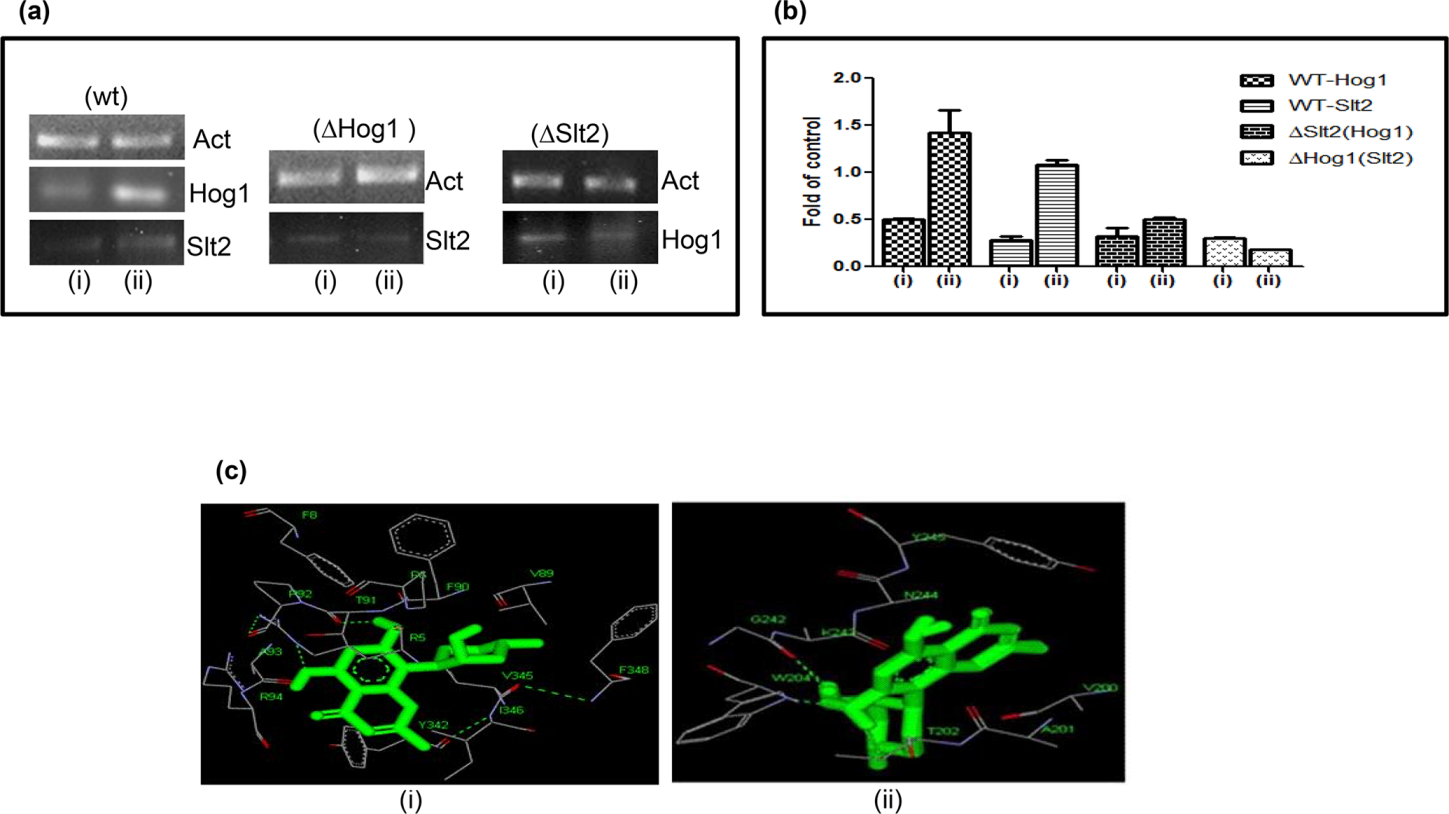
(a) RT-PCR analysis of *Slt2* and *Hog1* gene in budding yeast after drug treatment (i:Untreated control, ii: drug treated) (b) The expression of Slt2 and Hog1 mRNA, expressed as the ratio of densitometric measurement of the sample to the corresponding internal control (β-actin) (i: Untreated control, ii: drug treated) (c) Docking studies of Rohitukine with human two different type of member of MAPK pathway. (i) p38 (Hog1 in *S*. *cerevisiae*) and (ii) ERK5 (Slt2 in *S*. *cerevisiae*).

The mRNA levels of *Slt2* and *Hog1* were found to be 3.7 and 2.8 fold increased respectively in WT strain treated with Rohitukine when compared to that of control group (Fig 4A). However mRNA expression of *Slt2* gene in ΔHog1 strain and expression of *Hog1* gene in ΔSlt2 strain remained un-affected after Rohitukine treatment. Fig 4B depicts the fold changes in mRNA levels within different treatment groups normalized against that of control. The selective increase of Slt2 and Hog1 in wild type conditions and not in the knockouts of either gene may be a result of cross-talks between different MAPK pathways which are very common [42]. The over expression of *Hog1* gene after Rohitukine treatment may be dependent on the presence of *Slt2* gene or vice-versa.

Zymolyase activates both MAPKs and Slt2 activation depends on the Sho1 branch of the HOG pathway. Both MAPK pathways are essential for cell survival in presence of stress because mutant strains deficient in different components of both pathways are hypersensitive to zymolyase [43]. Thus, a sequential activation of two MAPK pathways may be required for cellular adaptation to stress condition and cell wall damage after the Rohitukine treatment.

From previous studies it is known that Hydroxyurea treatment increases phosphorylation of Slt2 MAP kinase [28]. Slt2 is responsible for cell wall integrity and is activated by cell wall damage, so it might be the possible reason for hypersensitivity of slt2 mutant to drug treatment. Recent studies have implicated the role of Hog1 MAPK in mediating tolerance to a variety of stress conditions including osmotic, oxidative, heat, arsenic, and citric acid stress [44]. A study by Azad et al examined the requirement for a functional HOG pathway to cope with curcumin (100 μM) induced stress [45].

Our docking studies revealed that catalytic domain of P38 (Hog1) interacts with Rohitukine through the nine amino acid residues namely VAL89, ARG5, ARG94,VAL345, ILE346, PHE348, PHE8, PHE90, and ASP88, while in case of ERK5 (Slt2) interaction was found with 4 amino acid residues namely TYR245, VAL200, TYR199 and ASN244 (Fig 4C). The free binding energy and estimated inhibition constant (ki) for the ‘Rohitukine-P38 domain interaction’ was determined as -6.47 Kcal/mol and 18.18uM respectively; while the same for ‘Rohitukine- ERK5 domain interaction’ was found to be -6.31 Kcal/mol, 23.6 u Mol respectively.

### Rohitukine induced cytotoxic effects by ROS generation in A549 cell line

**Fig 5:**
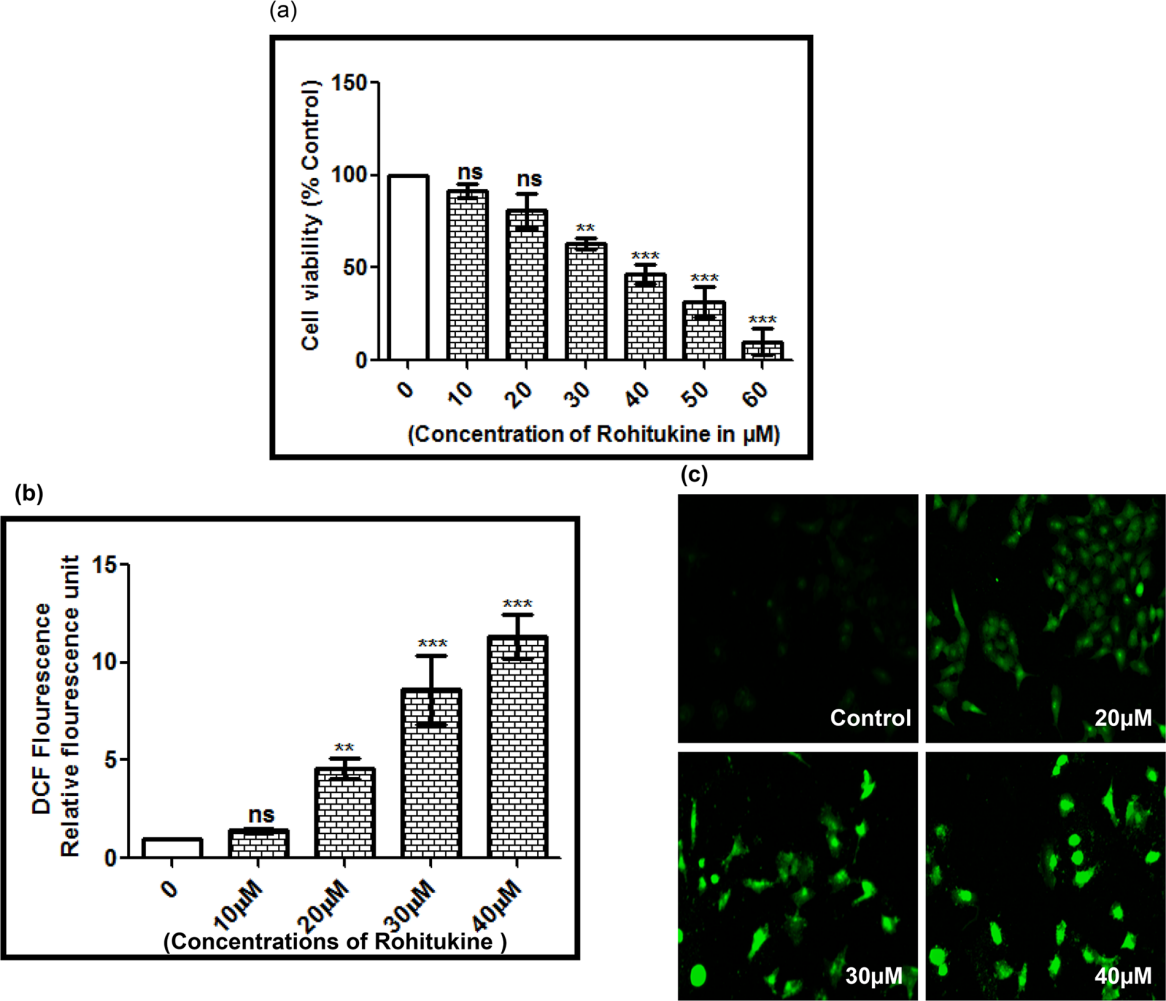
Rohitukine affected the percentage of viable and induced ROS in A549 cells. (a) Cell viability was determined using the MTT assay. Cells (1 × 10^4^ cells/well; 96 well plates) were plated in DMEM F12 medium + 10% fetal bovine serum (FBS) with 0, 10, 20, 30, 40, 50 and 60 μM for 24 hrs, (b) ROS generation was assessed in terms of relative fluorescence units using 10 mM DCFH-DA in A549 cells after 24 hrs exposure to Rohitukine in black-bottomed 96-well plates and (c) Fluorescence micrographs of ROS generation at 20μM, 30μM, and 40μM Rohitukine concentrations in lung cancer cells obtained at 20X objective after 24 hrs of treatment. The results are represented as means ±S.D of three independent experiments. Nonsignificant (ns), * *P* < 0.05, ** *P* < 0.01, *** *P* < 0.001 versus 0 μM.

To examine the cytotoxicity of Rohitukine, A549 cells were treated with different doses of Rohitukine (10μM to 60μM) for 24 hrs and the viabilities of cells were determined using the MTT assay. As shown in Fig 5A, Rohitukine significantly reduced percentage of viable A549 cells in dose-dependent manner. Among all the tests, cells incubated with 40 μM Rohitukine for 24 hrs showed anti-proliferation effect, with cell viability decreased to 50% of the untreated control cells. All experiments were carried out at dose below IC_50_ value.

The crude methanol extract of *F*. *proliferatum* that is the source of Rohitukine shows cytotoxicity against HCT-116 and MCF-7 human cancer cell lines (IC_50_ = 10 μg/ml for both cancer cell lines) [25]. Pure Rohitukine from stem barks of *D*. *binectariferum* was subjected for anticancer activity in different lung and ovarian carcinoma cells. IC_50_ value for ovarian carcinoma cells SKOV3 was found to be 20μM and for breast cancer cells T47D, MDAMB273, MCF7 was found to be 50μM, 3μM, 15μM respectively [46].

Flavopiridol, a semi synthetic derivative of Rohitukine exhibited anticancer activity by inhibiting cell cycle dependent kinases (CDKs) [47] Rohitukine possessed anti-estrogenic effect in female Sprague-Dawley rats [48] and compound that show antiestrogenic activity could also have antiproliferative effect on breast cancer cell by cell cycle arrest, including decreased cyclin Dl expression [49].

After determination of cytotoxic effect of Rohitukine in lung cancer cell we checked the effect of drug on ROS generation as in sight of earlier finding that copious chemical stimuli prompt apoptosis via ROS generation [50]. We employed H2-DCF-DA (specific fluorescence probes) staining to examine ROS generation in A549 cells after 24 hrs of Rohitukine (10μM to 40 μM) treatment. Significant elevation in ROS levels could be observed at all the tested doses (Fig 5B). Fluorescence micrographs of H2-DCF-DA stained cells further confirmed the above fluorometric findings (Fig 5C). Data from the current study revealed that Rohitukine induces oxidative stress in A549 cells.

Oxidative stress by ROS is a stimulator of numerous cell responses, such as apoptosis in various mammalian cells [50]. Flavopiridol have been shown to alter the redox status of leukemic cells and mediated apoptosis is dependent upon generation of radical oxygen species [51].

This study showed that Rohitukine acts as a ROS generator to trigger cell death in lung cancer cells, supporting its utility as a cytotoxic therapeutic agent [52].

### Rohitukine altered the apoptosis-associated protein levels in A549 cells

**Fig 6:**
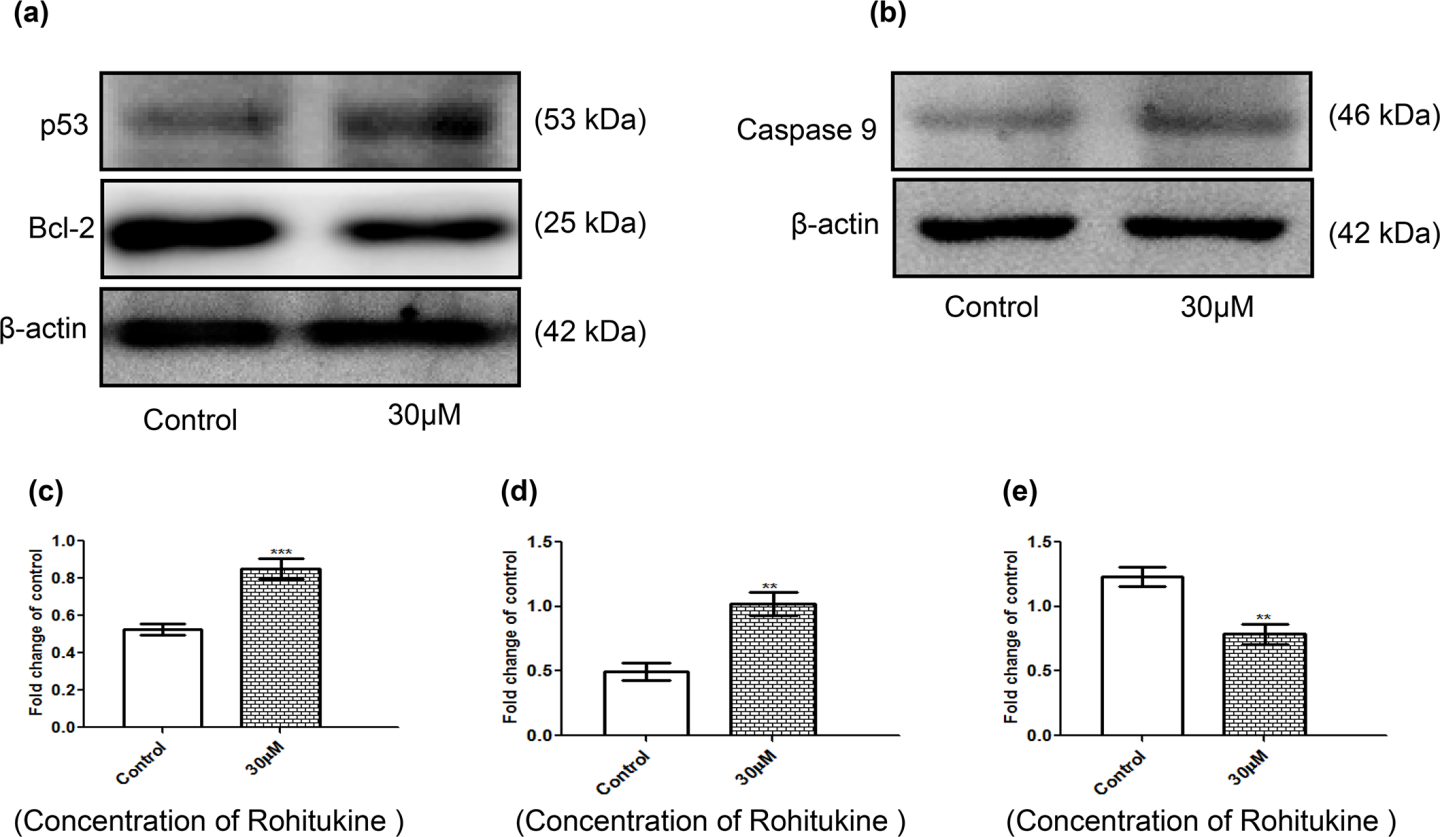
Rohitukine affected the apoptosis-associated protein levels in A549 cells. **Cells were treated with Rohitukine at 30** μ**M for 24hrs, and then the total proteins were prepared and determined as described in methods.** (a) The levels of proteins expression of p53 and Bcl-2 (b) proteins expression of caspase9 were estimated by Western blotting. Band intensities were calculated by densitometry and change in protein expression after Rohitukine treatment was calculated with respect to controls and expressed as fold change in graph. (c), (d) & (e) densitometry for p53, Bcl-2, and caspase9 blot respectively. Results were normalized to β-actin. The data are represented as means ±SD of three independent experiments (** *P* < 0.01 versus control).

Cells were exposed to 30 μM of Rohitukine for 24 hrs and then the total protein was prepared and western blot analysis was used to detected protein expression of p53, caspase9 and Bcl-2. β-actin was used as an internal loading control. These results are presented in Fig 6 which indicated that expression of p53 (Fig 6A) and caspase9 (Fig 6B) was increased, while the expression of Bcl-2 (Fig 6A) was decreased. The quantitative results showed that Rohitukine increased the protein levels of p53 and caspase9 by 0.40 folds (Fig 6C) and 0.23 (Fig 6D) folds respectively where as decreased the protein levels of Bcl-2 by 0.37 folds (Fig 6E) of the control level at 30 μM. Upregulation of proteins like, p53 caspase9 and down regulation of anti-apoptotic proteins like Bcl-2 gives insight into the mechanism of action followed by Rohitukine to induce the apoptosis.

Tumor suppressor gene TP53 gets activated during genotoxic stress and promotes cell cycle arrest by the activation of p21 leading to the activation of apoptosis [53]. Caspase-9 activation leads to apoptosis. The majority of cancer therapy initiate apoptosis through the caspase-9 activation, the modulation of caspase-9 expression may be exploited in designing new ways to control apoptosis in neurodegenerative or malignant diseases [54]. Bcl-2, an upstream effector molecule in the apoptotic pathway, has been recognized to be a potent negative regulator of apoptosis, and most cancers generally overexpress Bcl-2 [55]. MAPK pathways also regulate apoptosis and activation of p38 is generally associated with the induction of apoptosis. Berberine (a benzylisoquinoline alkaloid) significantly inhibited growth and induced cell cycle arrest of NSCLC cells (non small cell lung cancer cells) in a dose-dependent manner. It increased phosphorylation of p38 MAPK in a time-dependent and induced protein expression of tumor suppressor p53. The specific inhibitor of p38 MAPK (SB203580), and silencing of p38α MAPK by siRNAs, blocked the stimulatory effects of Berberine on protein expression of p53 [56].

However some studies suggested that Inhibitor of p38 MAPK suppressed the proliferation of cancer cells by induction of cell apoptosis through the caspase activation showing the pro-oncogenic function of p38 in colon cancer, and its inhibition would be a novel strategy for the prevention and treatment of colon cancer [57]. Map kinase inhibition by map kinase inhibitor (SB203580 and U0126) decrease the cell viability and induced apoptosis in human CNE2 cells (human nasopharyngeal carcinoma cell line) [58].

Induction of apoptosis by flavopiridol in human leukemia cells (U937) proceeds via the intrinsic, cytochrome c-related pathway (caspase9 activation), and is not dependent upon the extrinsic, procaspase-8-associated cascade causing caspase activation and initiation of the apoptotic cascade [59]. Further Flavopiridol potently down regulated the levels of several antiapoptotic proteins in B-CLL cells in vitro, However, expression of the pro-apoptotic proteins Bax and Bak was not significantly influenced by Flavopiridol [60].

The effect of Rohitukine on regulating the expression of apoptosis-related proteins further supported the observation that Rohitukine induced apoptosis in A549 cells.

*Dysoxylum binactariferum* stem bark as well as its major active constituent Rohitukine possesses diverse biological activities including anti-inflammatory, immunomodulatory, anti leishmanial and cancer activities. However, for the first time mechanism of action of anticancer activity of Rohitukine have been evaluated for budding yeast and lung cancer cell line as well.

Our study also used *S*. *cerevisiae* which is a powerful tool for studying the effects of drug on eukaryotic cells. We showed that Rohitukine enhances oxidative stress which leads to induction of apoptosis. The pattern of apoptosis induction is differential in WT and MAP kinase gene knockout strains indicating a critical role of MAPK in induction of cell death after Rohitukine treatment. The results of our study provide first evidence of the role of MAPK pathway in mediation of anticancer activity of Rohitukine by triggering an apoptotic phenotype in *S*. *cerevisiae*. It is shown to impart its anti-cancer property by induction of ROS which is a key marker of apoptosis, upregulation of proapoptotic protein (p53) and down regulation of antiapoptotic protein (Bcl-2). This proposed mechanism might have broad implications in cancer therapeutics.
